# Celerina

**Published:** 1882-08-20

**Authors:** 


CELERINA.—Read the new advertisement of this excellent preparation commencing with this number of our Journal.

THE American Pharmaceutical Association will hold its thirtieth annual meeting at Niagara Falls, commencing September 12th.

